# Supplementary figures and images for: Sir2 and Fun30 regulate ribosomal DNA replication timing via MCM helicase positioning and nucleosome occupancy
Source: eLife. 2025 Jan 20;13:RP97438. doi: 10.7554/eLife.97438 (PMC11745493; doi:10.7554/eLife.97438)

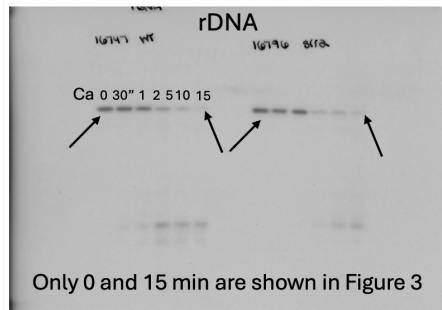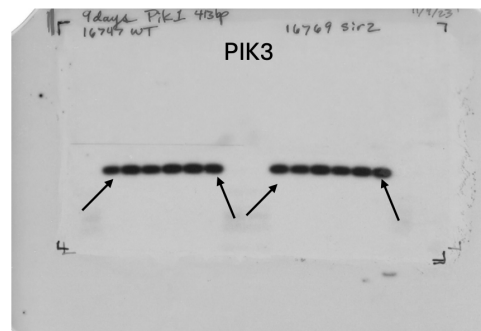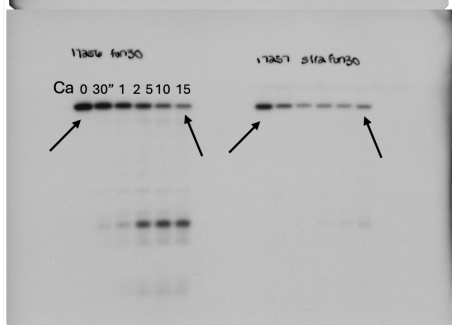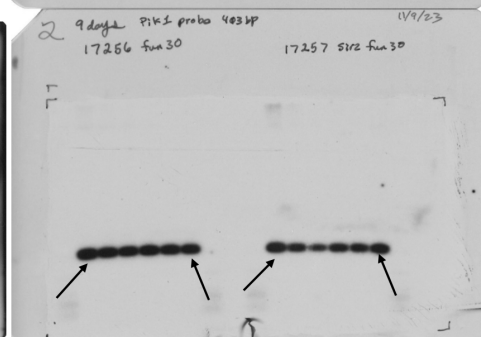

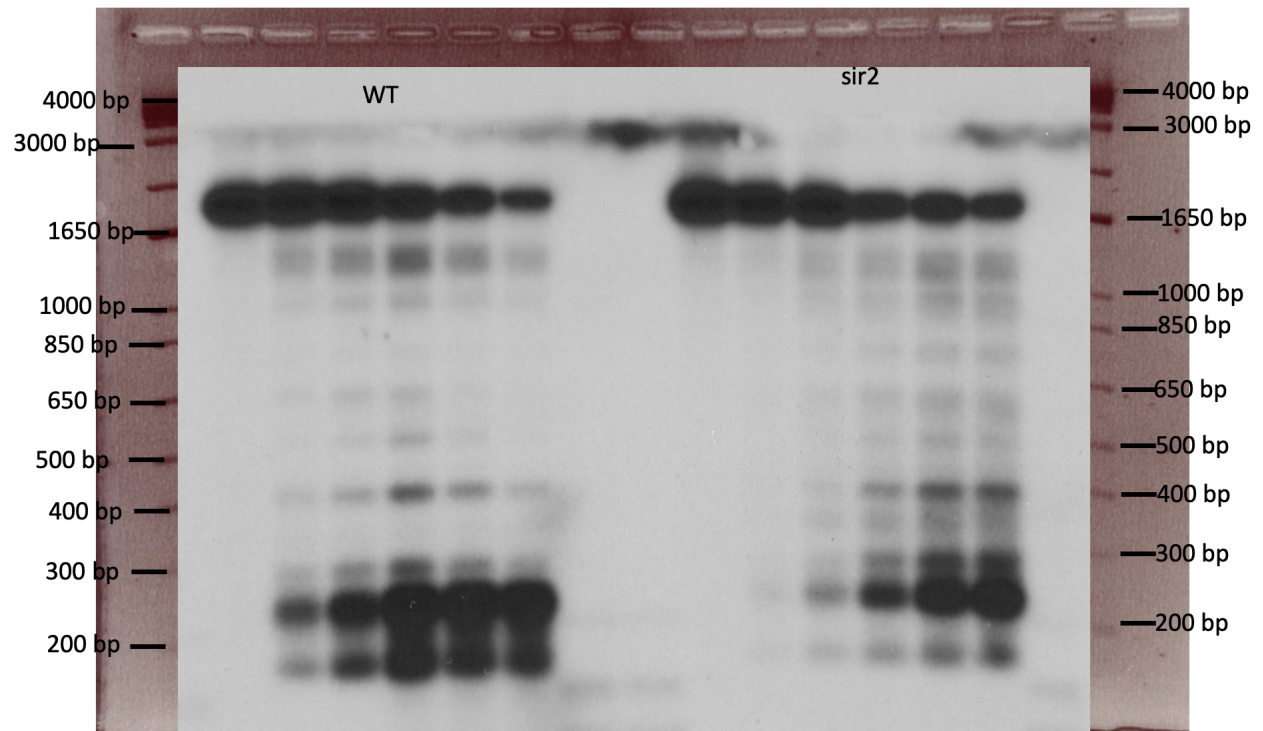

Southern blots overlayed with size markers.

Supplement: Figure 3—source data 1. [file elife-97438-fig3-data1.zip › Figure_3_source_data_1Revised.pdf]

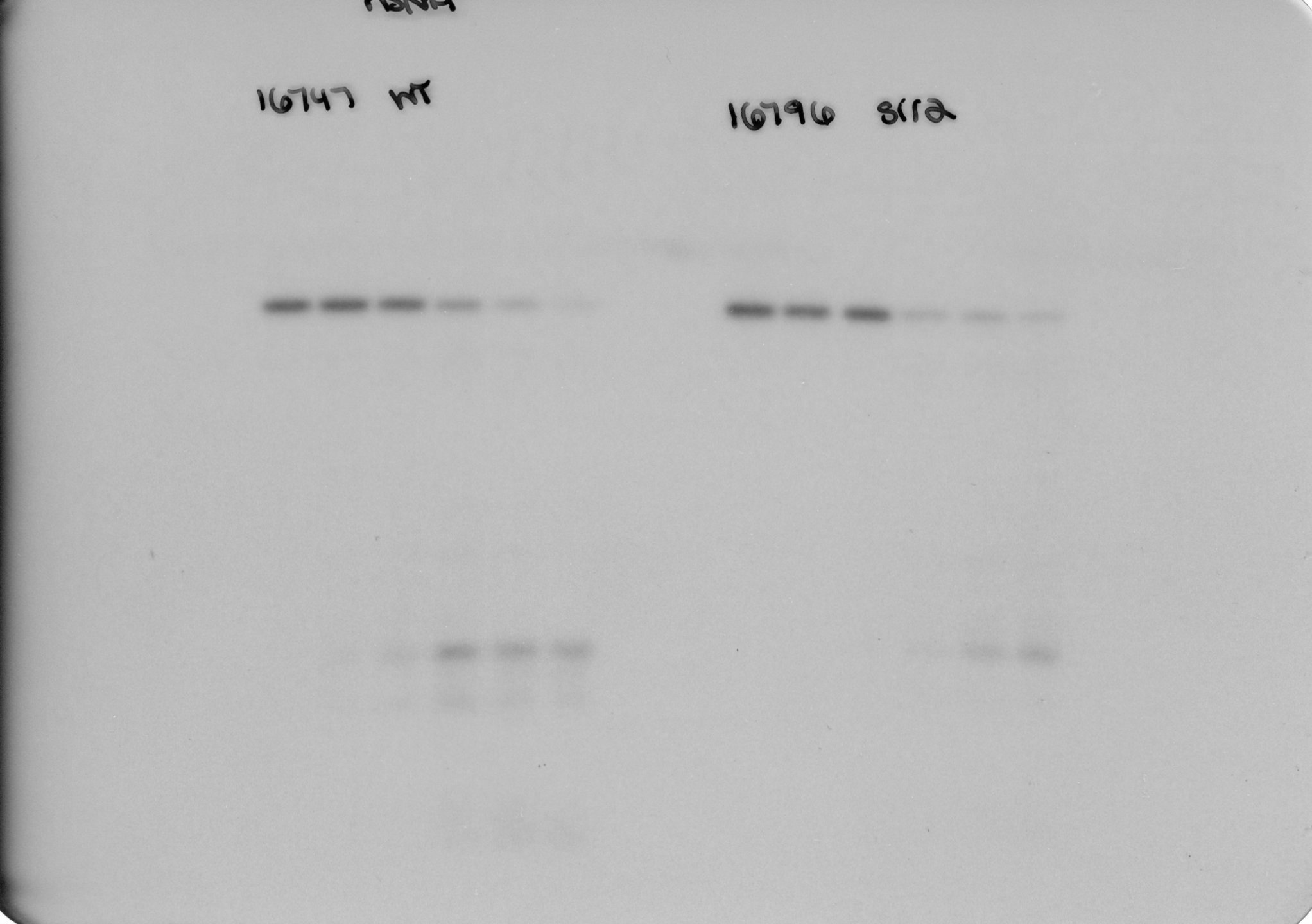

Supplement: Figure 3—source data 2. [file elife-97438-fig3-data2.zip › Figure_3_source_data_2_revised/rDNA_Probe_for_wt_sir2.tif]

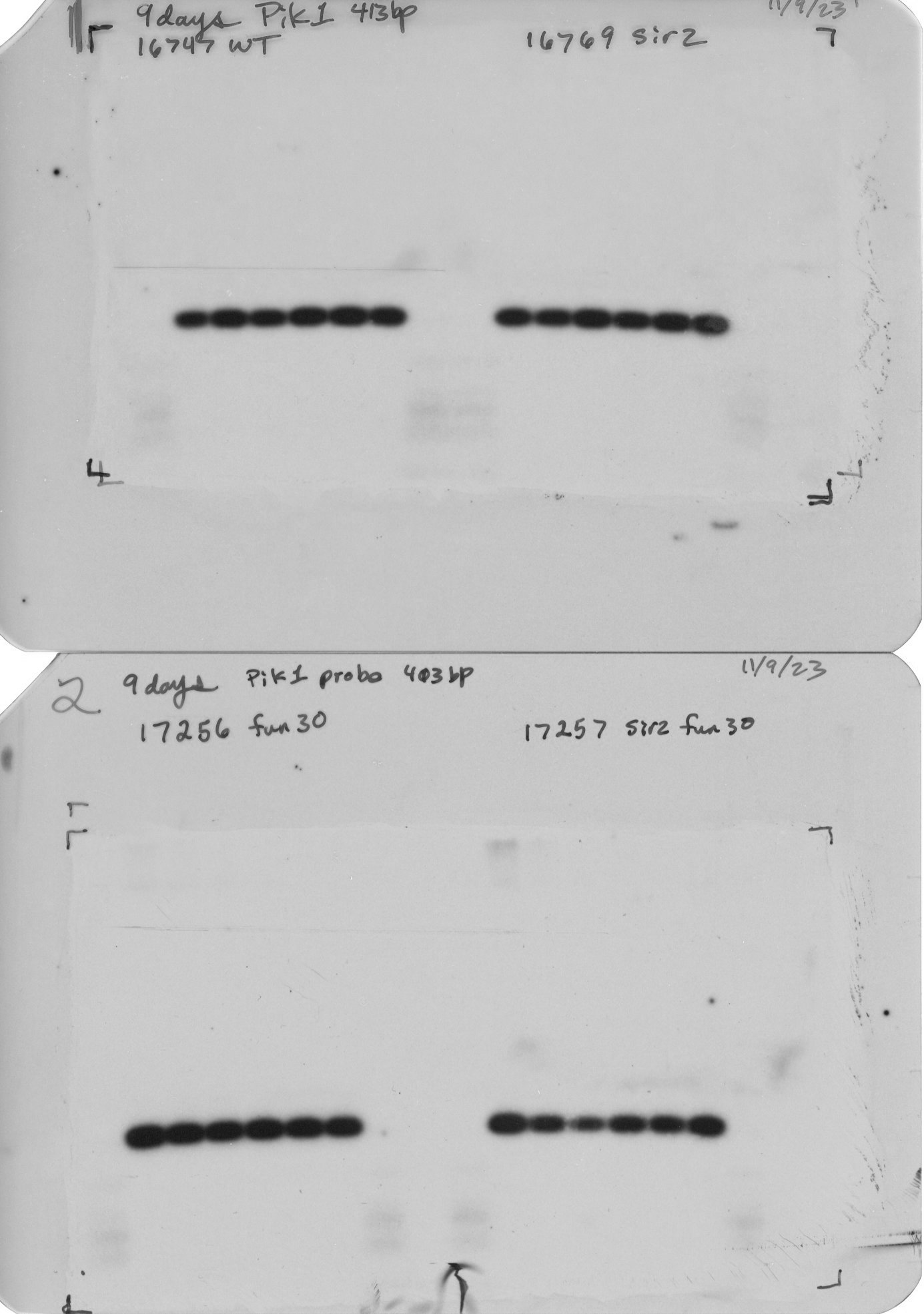

Supplement: Figure 3—source data 2. [file elife-97438-fig3-data2.zip › Figure_3_source_data_2_revised/PIK1_probe_all_genotypes.tif]

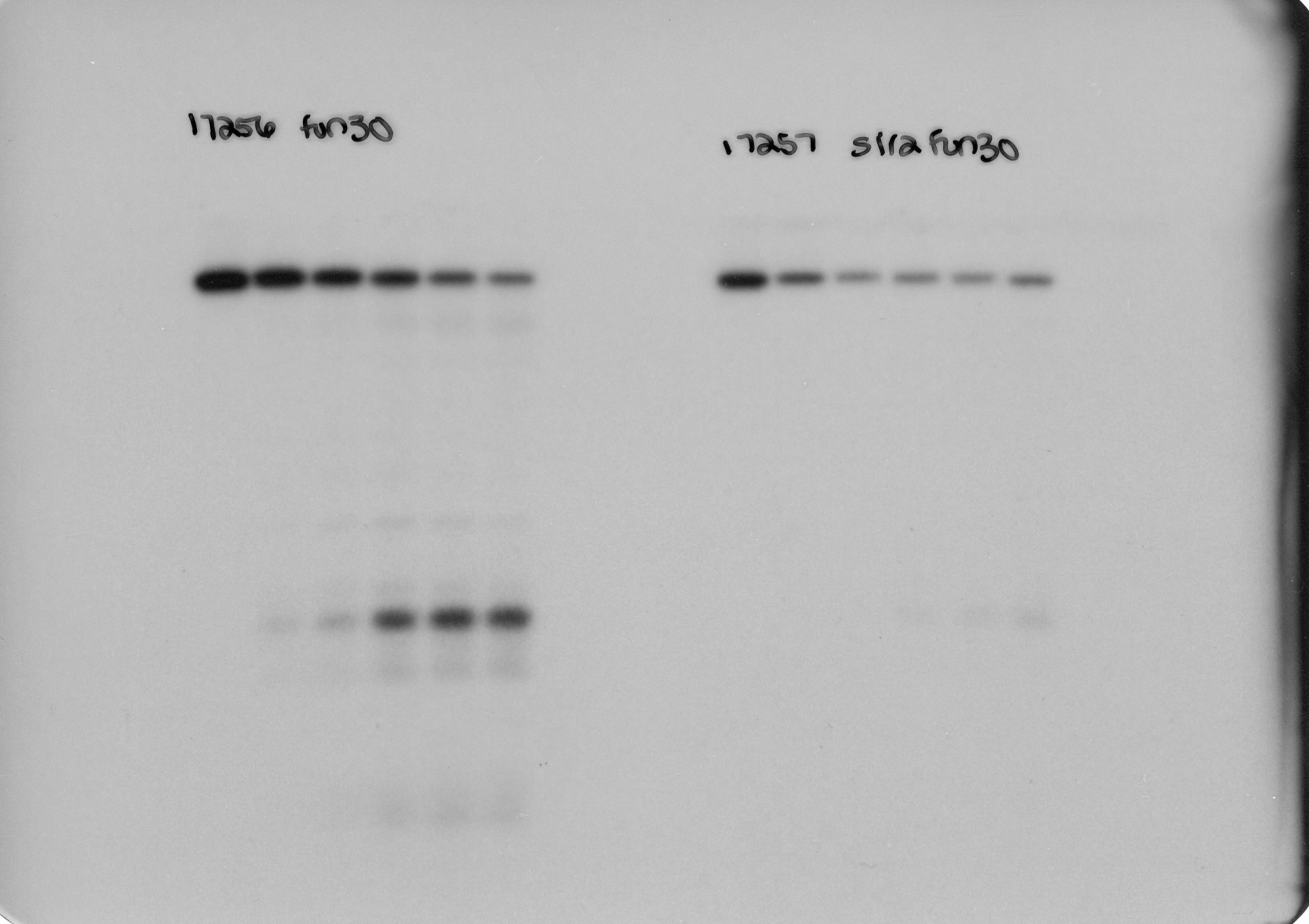

Supplement: Figure 3—source data 2. [file elife-97438-fig3-data2.zip › Figure_3_source_data_2_revised/rDNA_probe_fun30_sir2fun30.tif]

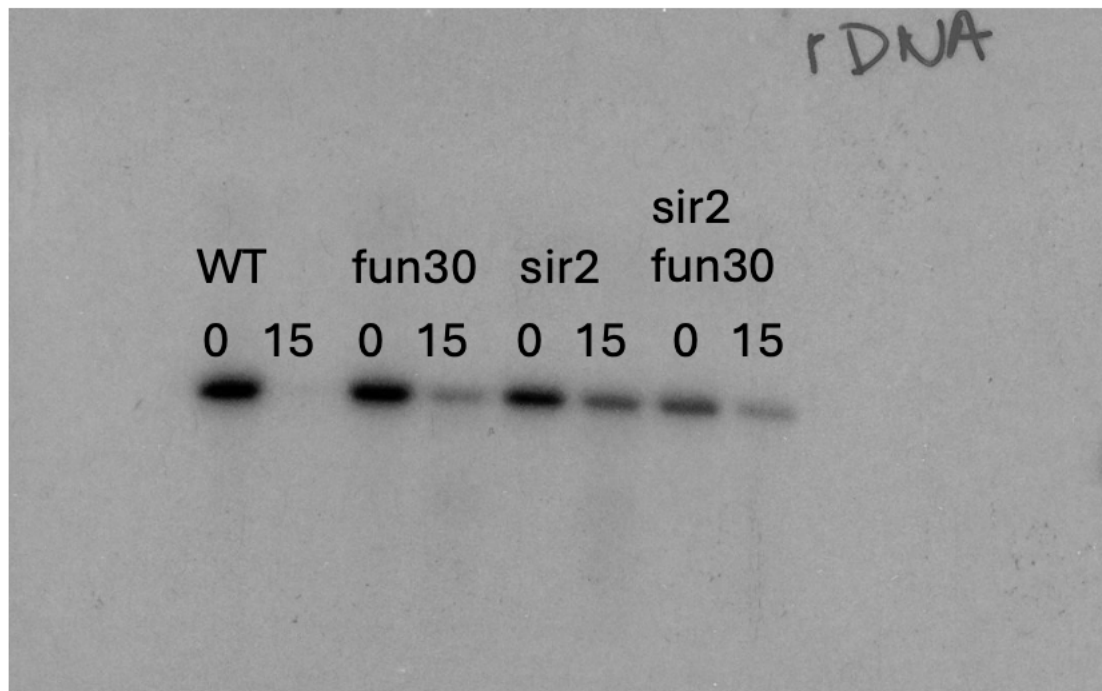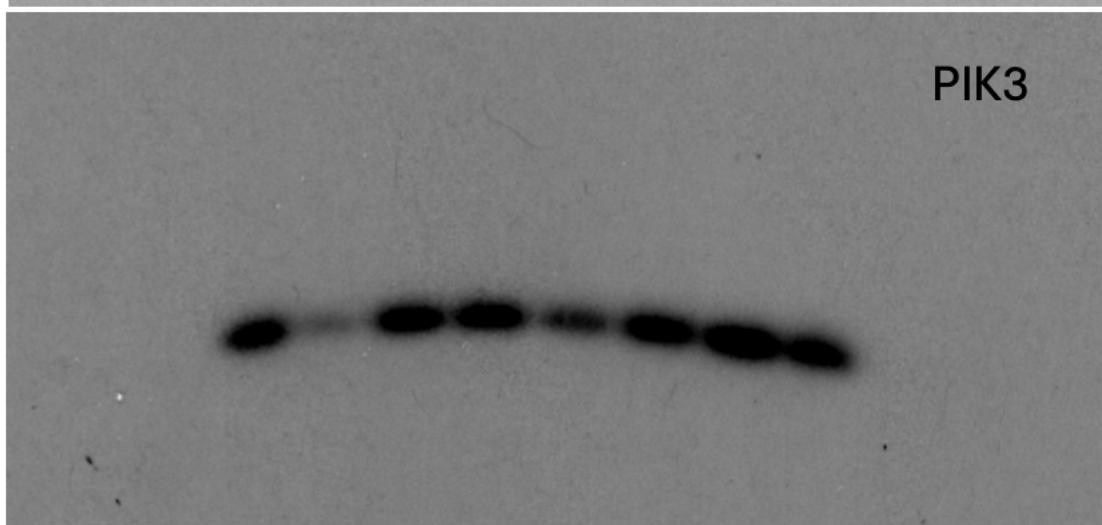

Supplement: Figure 3—figure supplement 1—source data 1. [file elife-97438-fig3-figsupp1-data1.zip › Figure 3_figure supplement 1_source_data_1/Figure_3_Suplemental-Figure 1 source_data.pdf]

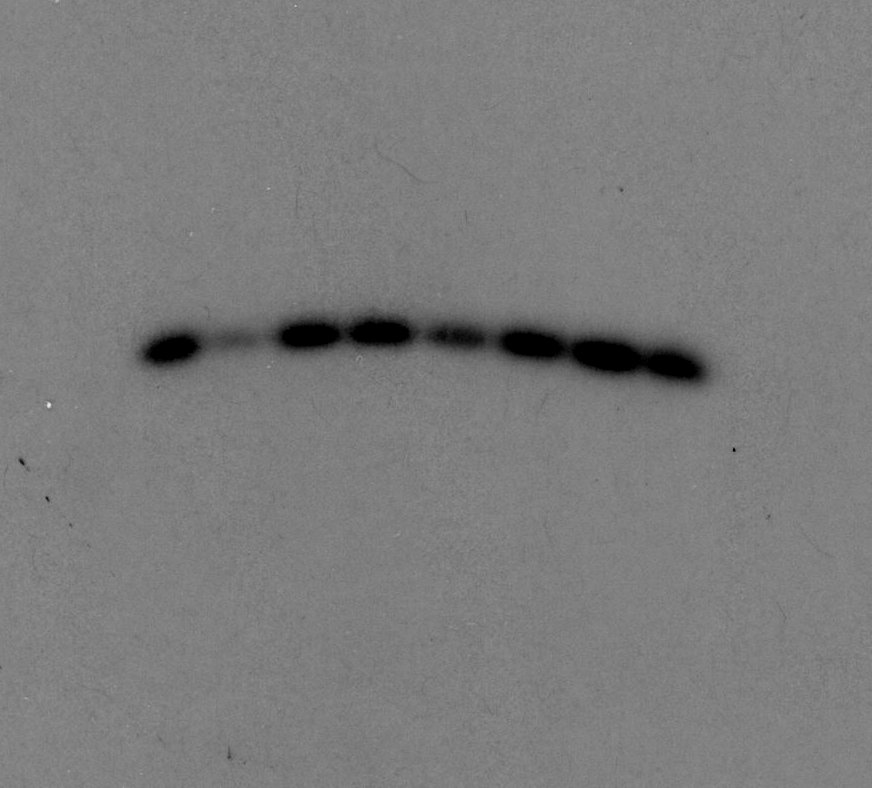

Supplement: Figure 3—figure supplement 1—source data 2. [file elife-97438-fig3-figsupp1-data2.zip › Figure 3_figure supplement 1_source_data_2/PIK3_probe_replica_Figure3_Figure supplement 1.tif]

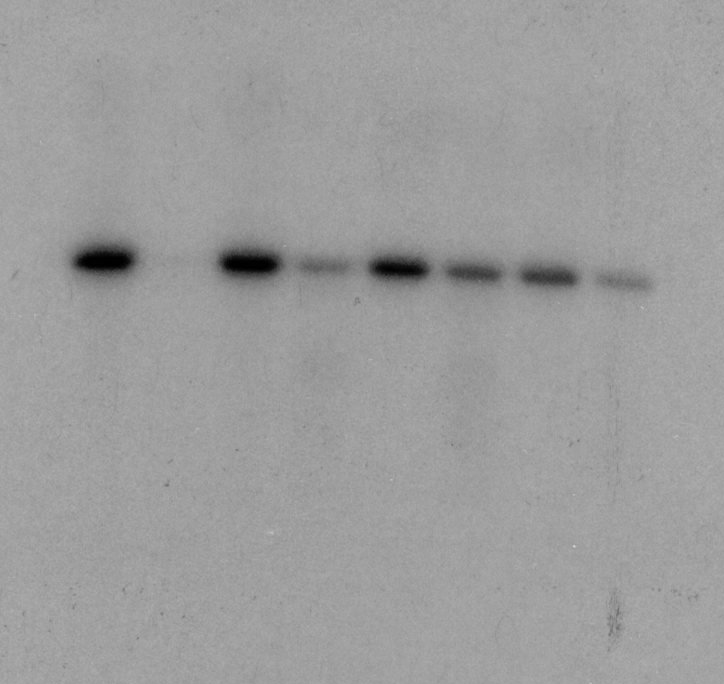

Supplement: Figure 3—figure supplement 1—source data 2. [file elife-97438-fig3-figsupp1-data2.zip › Figure 3_figure supplement 1_source_data_2/rARS_probe_Figure3_Figure supplement 1.tif]

Replica 1

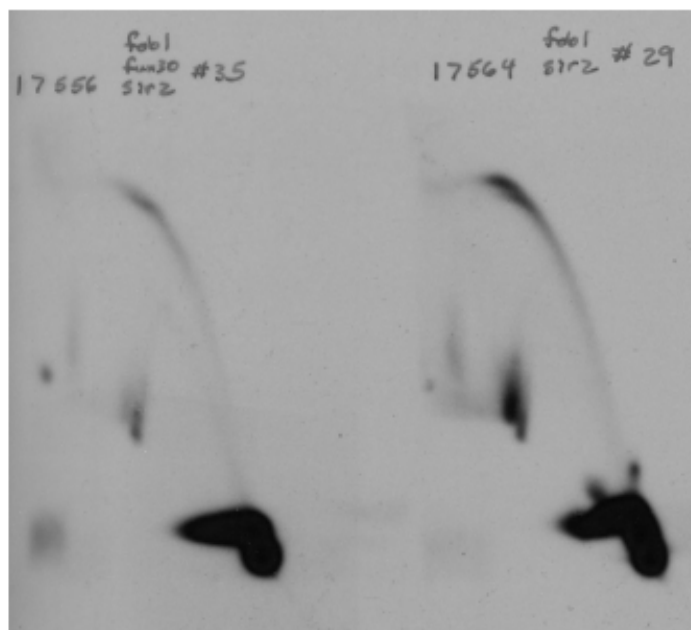

Replica 2

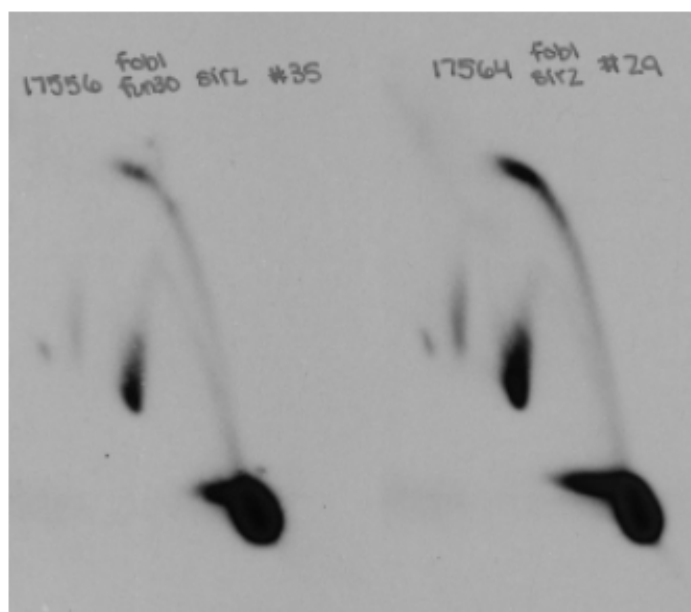

Supplement: Figure 4—source data 1. [file elife-97438-fig4-data1.zip › Figure 4 Source Data 1/Figure 4 C-source-data.pdf]

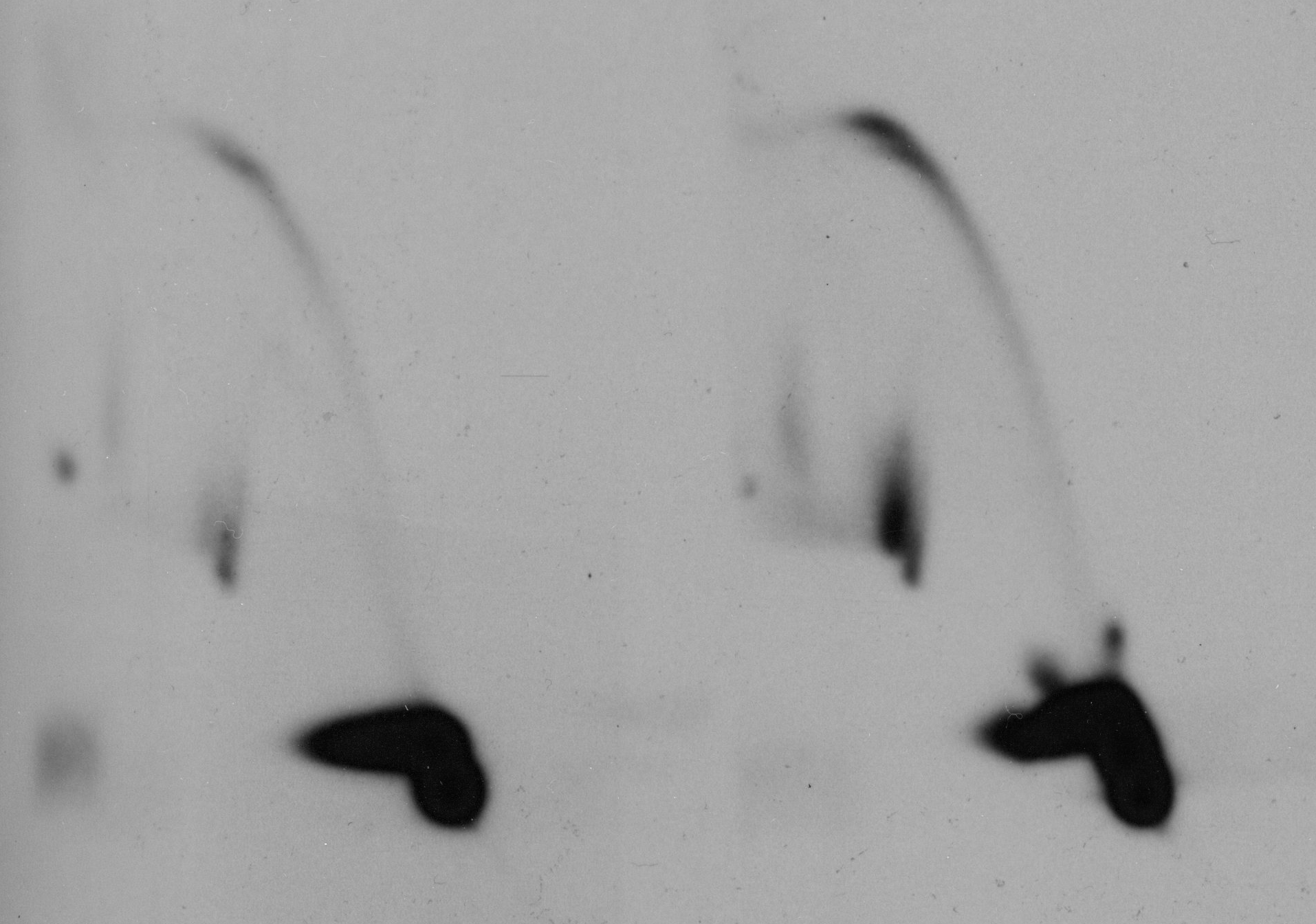

Supplement: Figure 4—source data 2. [file elife-97438-fig4-data2.zip › Figure 4 Source Data 2/Figure_4C_Replica1.tif]

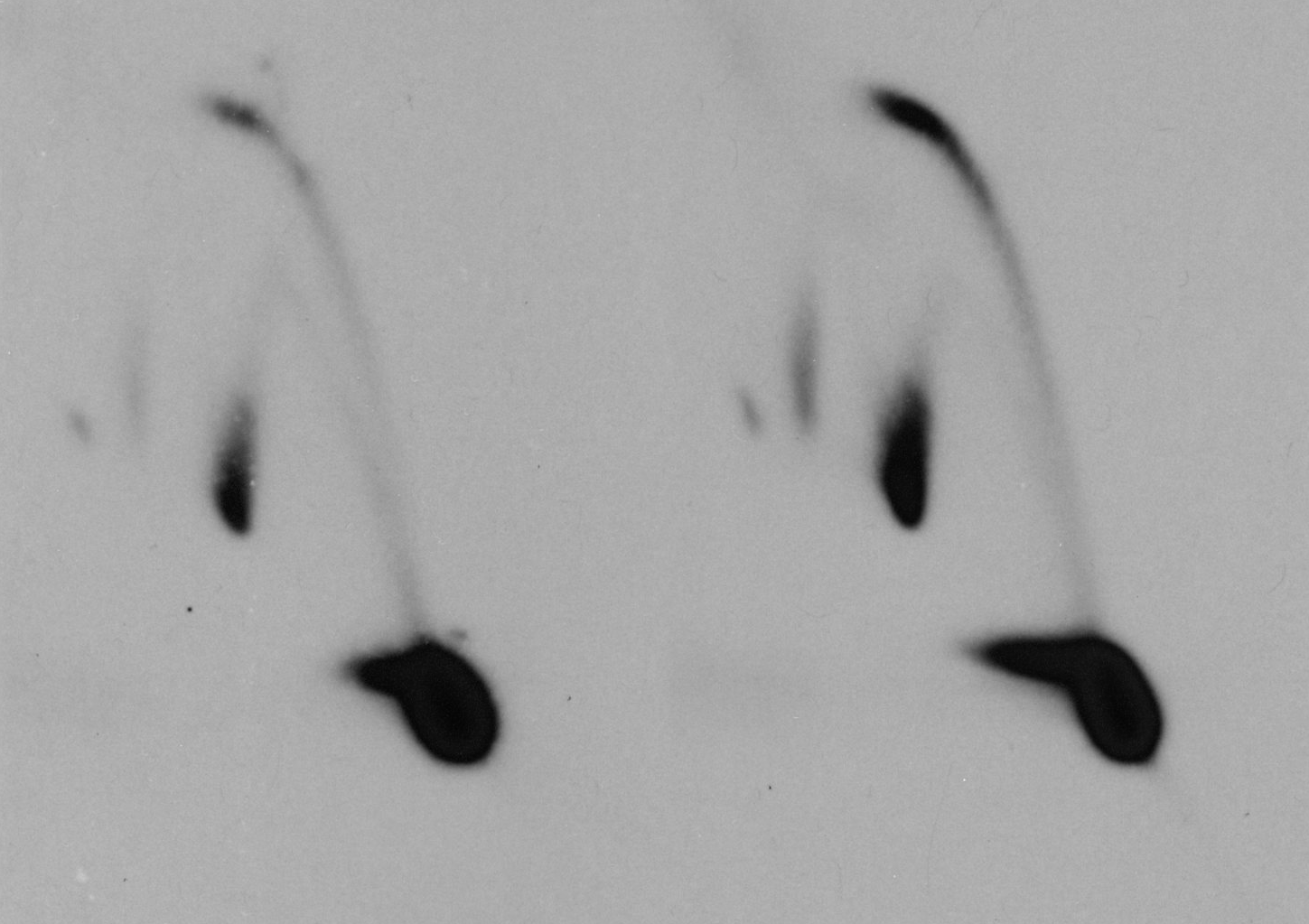

Supplement: Figure 4—source data 2. [file elife-97438-fig4-data2.zip › Figure 4 Source Data 2/Figure_4C_Replica2.tif]

**ARS305**  
**WT**

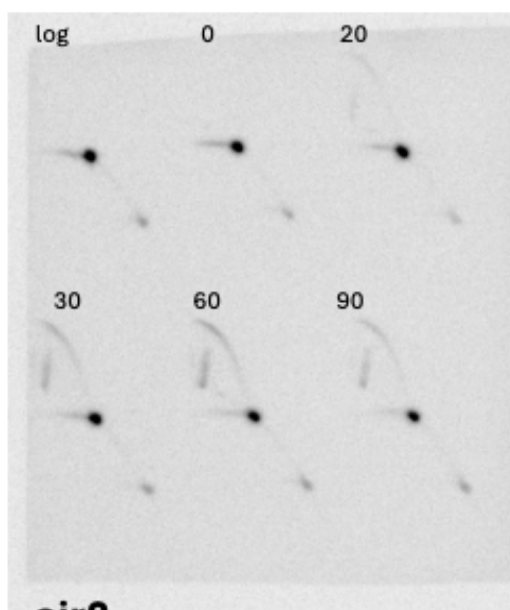

**sir2, fun30**

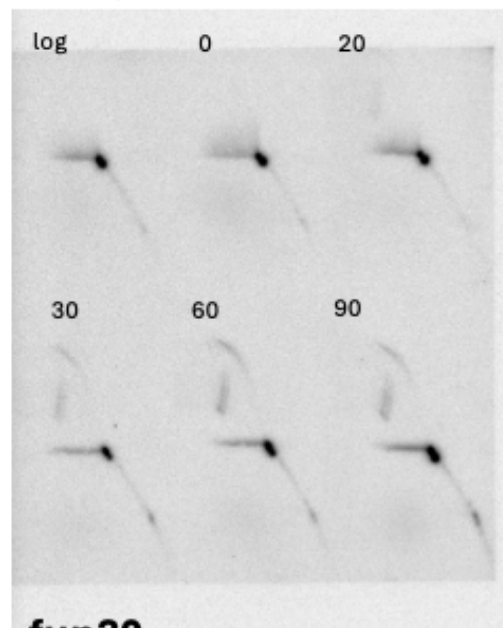

**sir2**

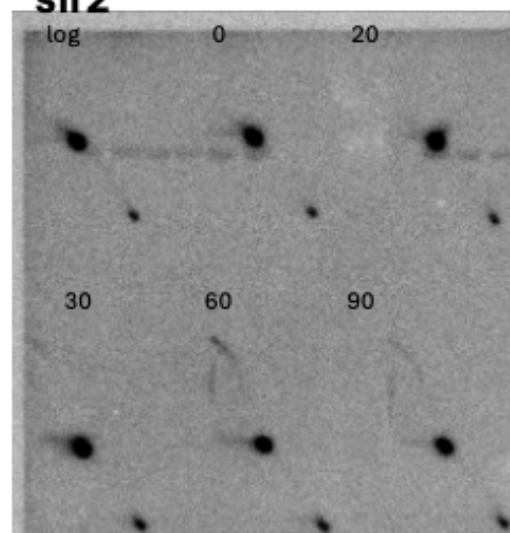

**fun30**

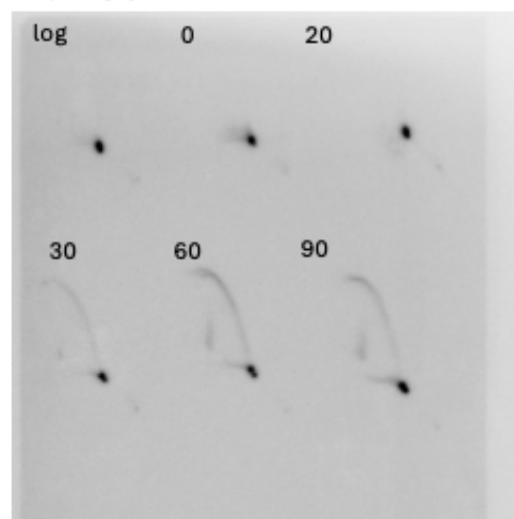

Supplement: Figure 4—source data 4. [file elife-97438-fig4-data4.zip › Figure 4 Source Data 4/ARS305_Figure_4E.PDF]

## rDNA

wt

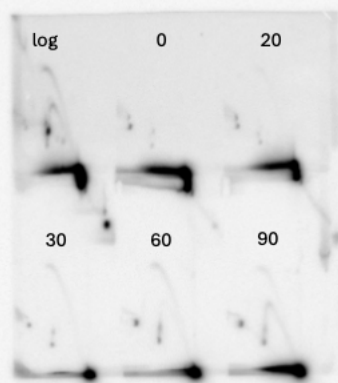

sir2 fun30

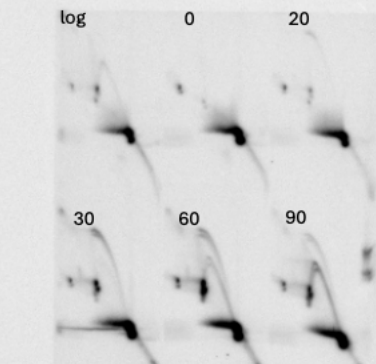

sir2

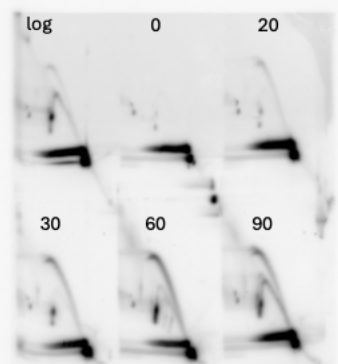

fun30

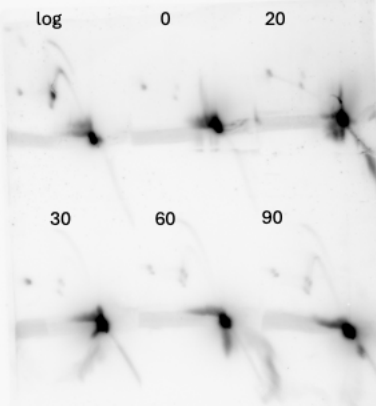

Supplement: Figure 4—source data 4. [file elife-97438-fig4-data4.zip › Figure 4 Source Data 4/rARS_Figure_4D.pdf]

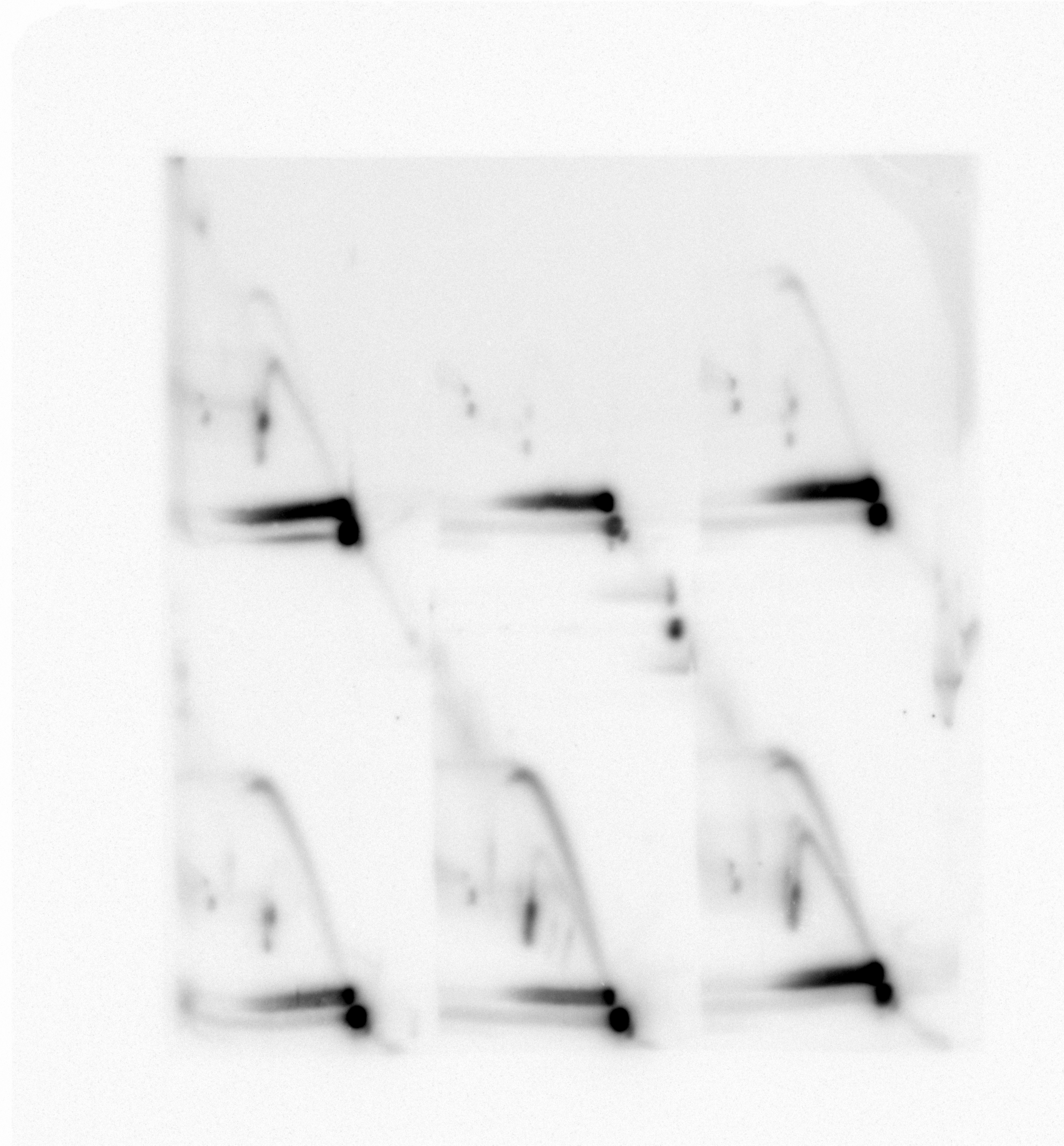

Supplement: Figure 4—source data 5. [file elife-97438-fig4-data5.zip › Figure 4 Source Data 5/rARS_probe_sir2.tif]

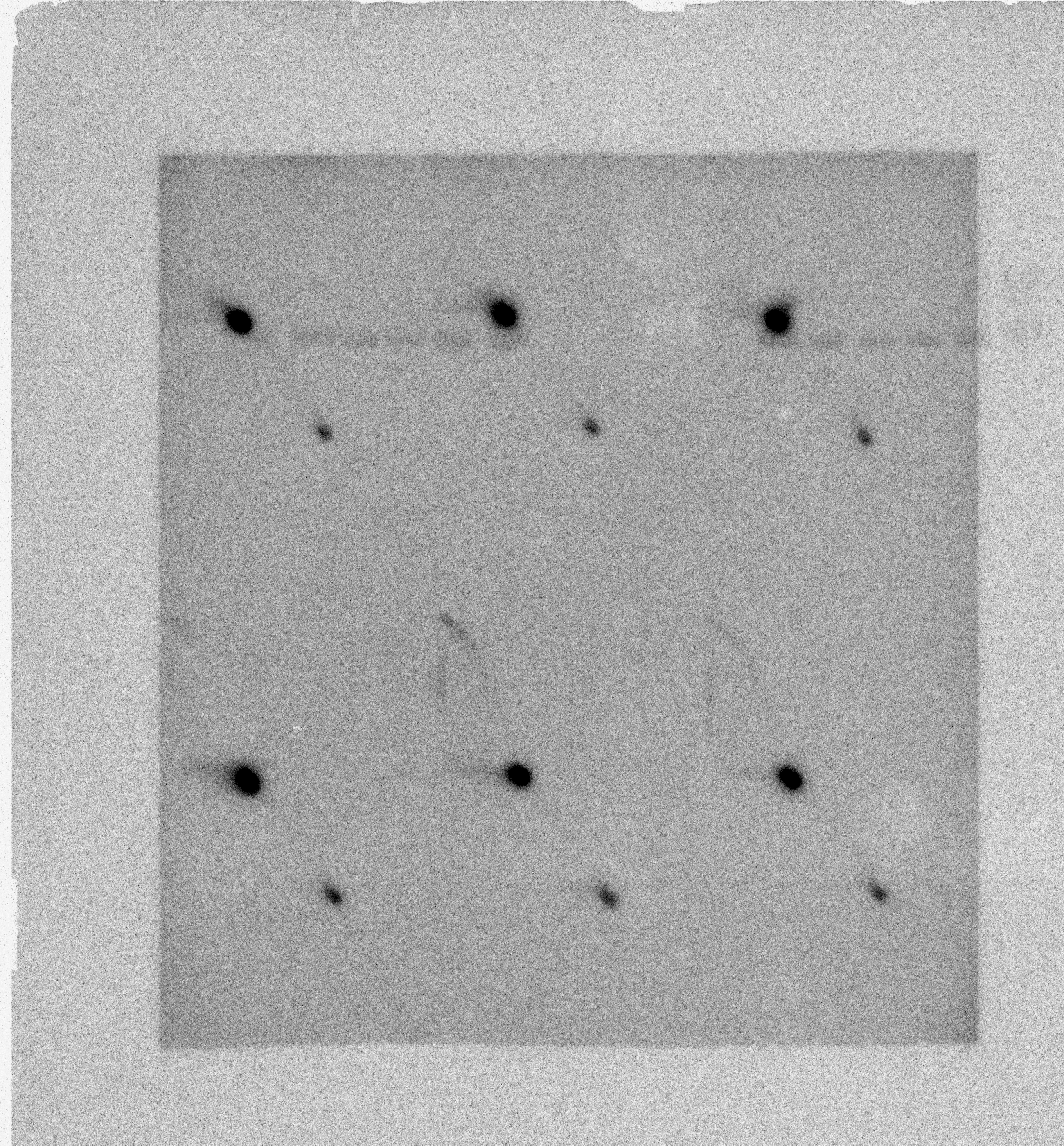

Supplement: Figure 4—source data 5. [file elife-97438-fig4-data5.zip › Figure 4 Source Data 5/ARS305_sir2.tif]

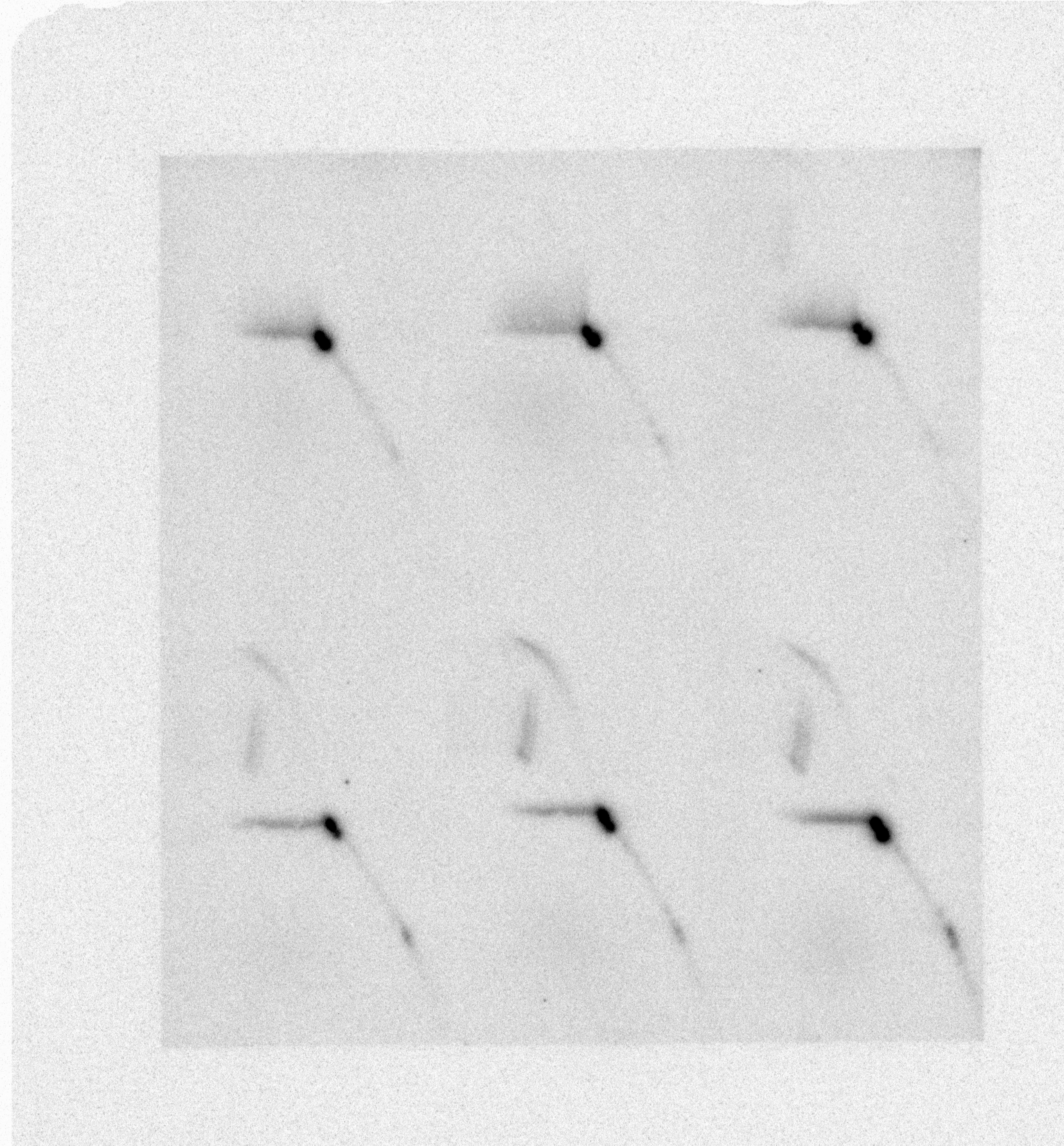

Supplement: Figure 4—source data 5. [file elife-97438-fig4-data5.zip › Figure 4 Source Data 5/ARS305_sir2 fun30.tif]

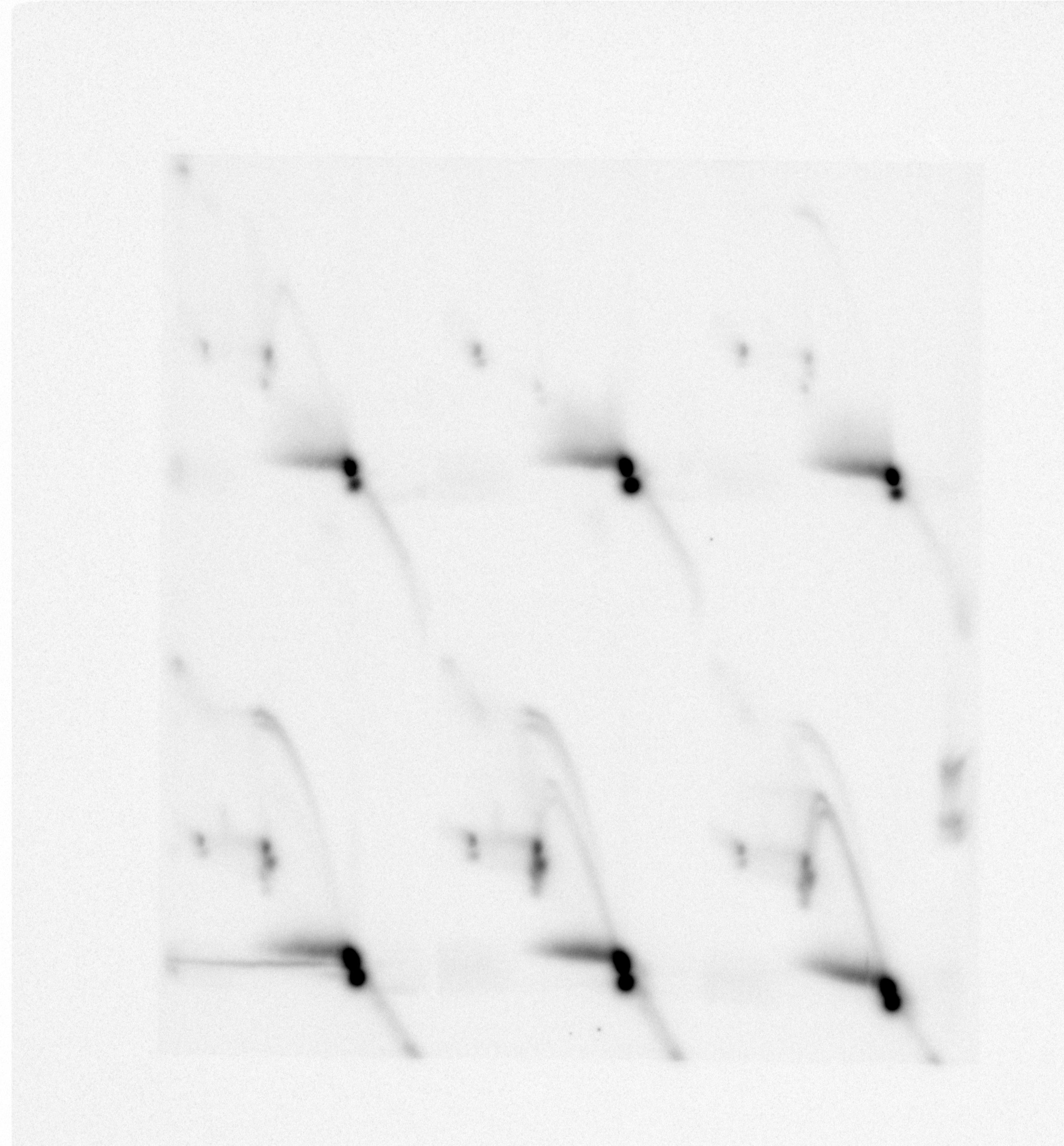

Supplement: Figure 4—source data 5. [file elife-97438-fig4-data5.zip › Figure 4 Source Data 5/rARS_sir2fun30.tif]

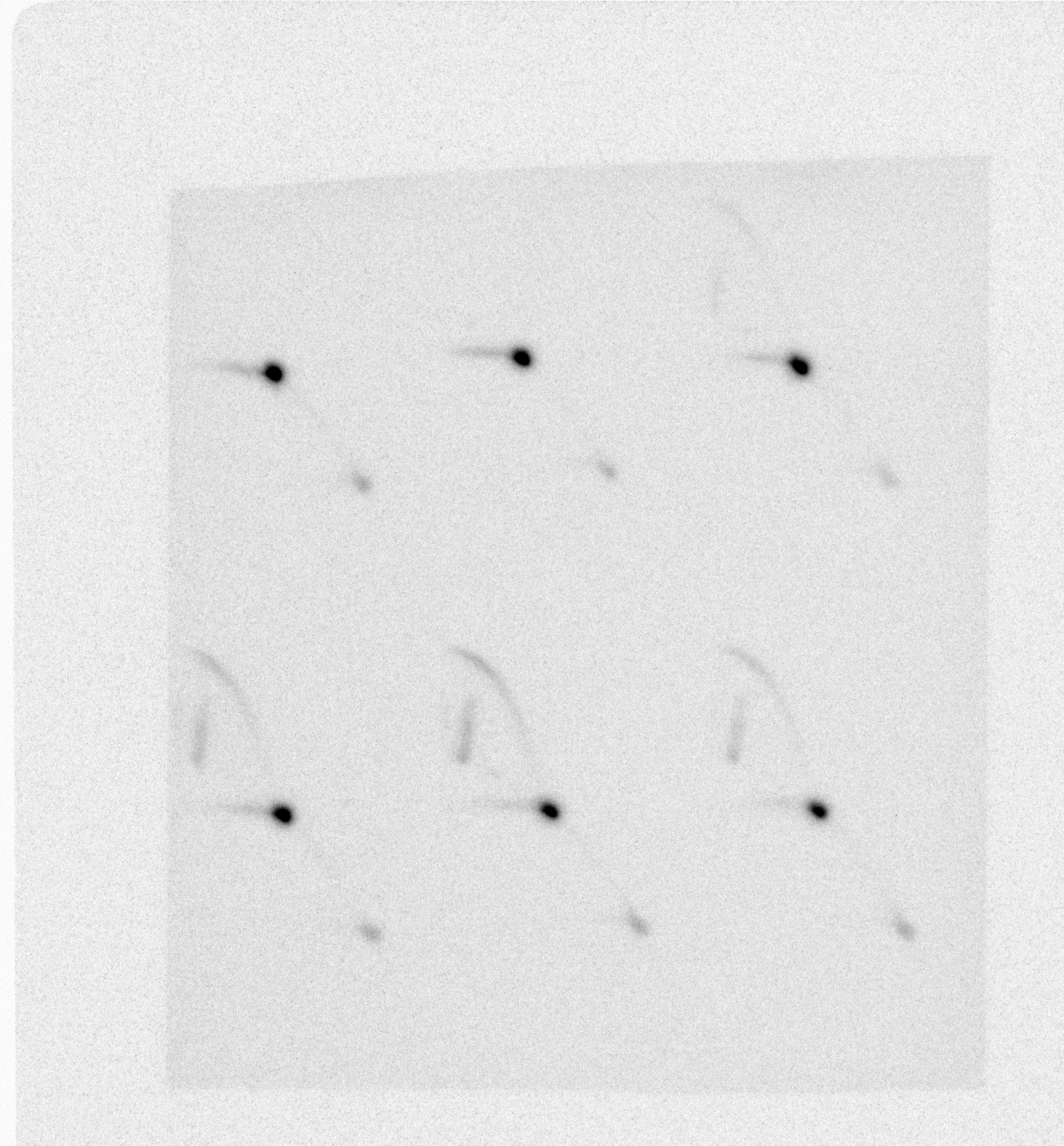

Supplement: Figure 4—source data 5. [file elife-97438-fig4-data5.zip › Figure 4 Source Data 5/ARS305_WT.tif]

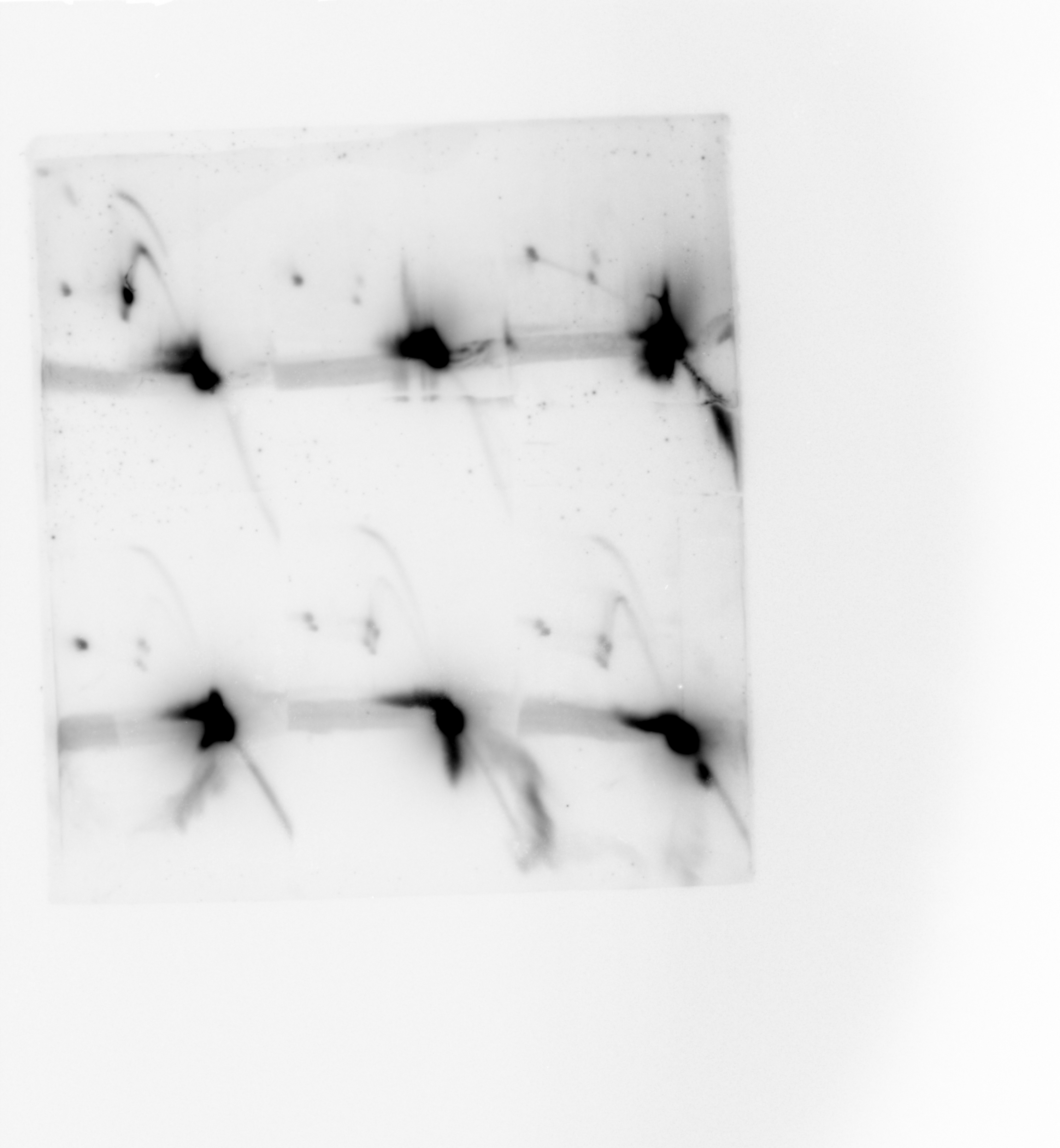

Supplement: Figure 4—source data 5. [file elife-97438-fig4-data5.zip › Figure 4 Source Data 5/rARS_fun30.tif]

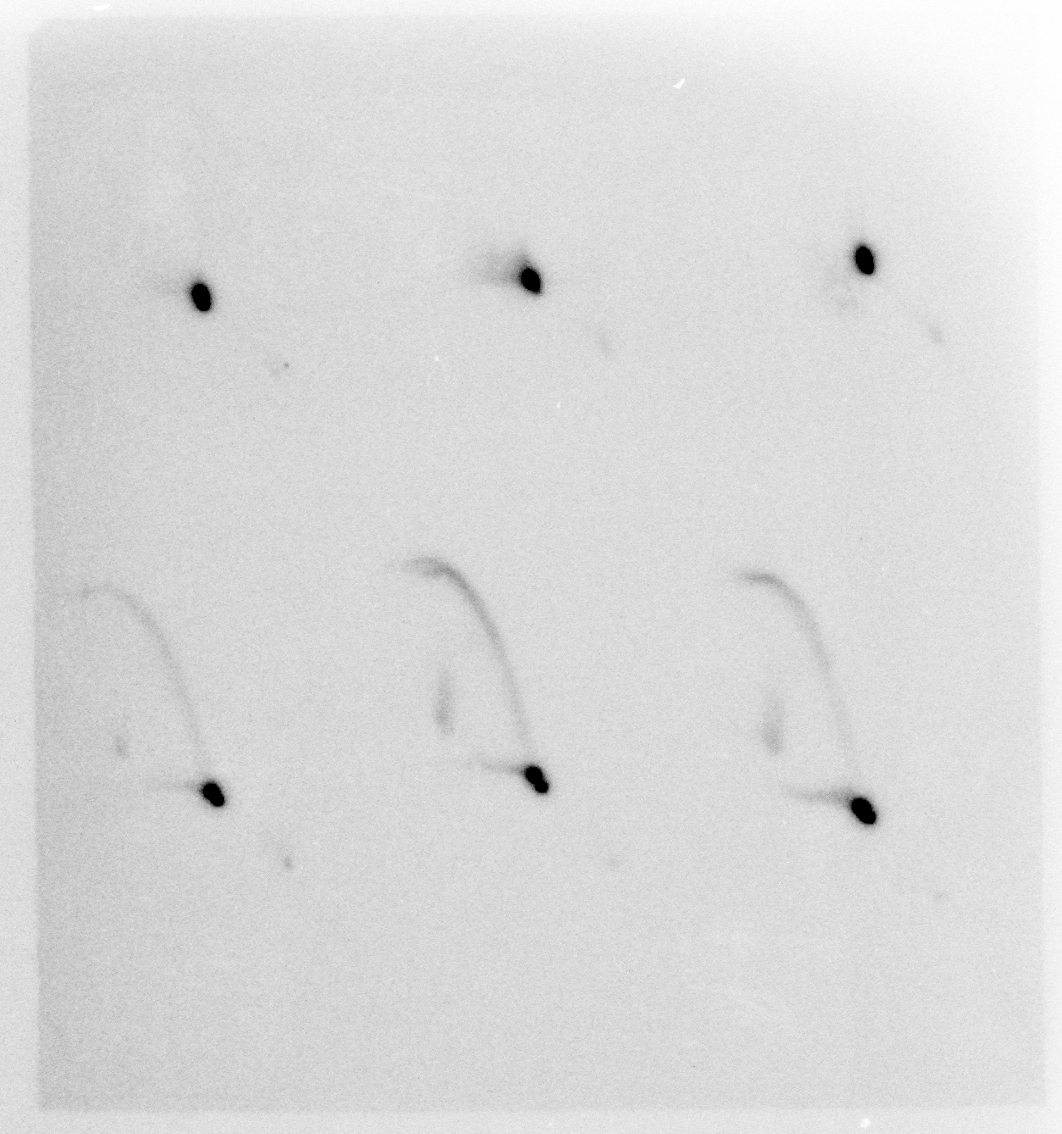

Supplement: Figure 4—source data 5. [file elife-97438-fig4-data5.zip › Figure 4 Source Data 5/ARS305_Fun30.tif]

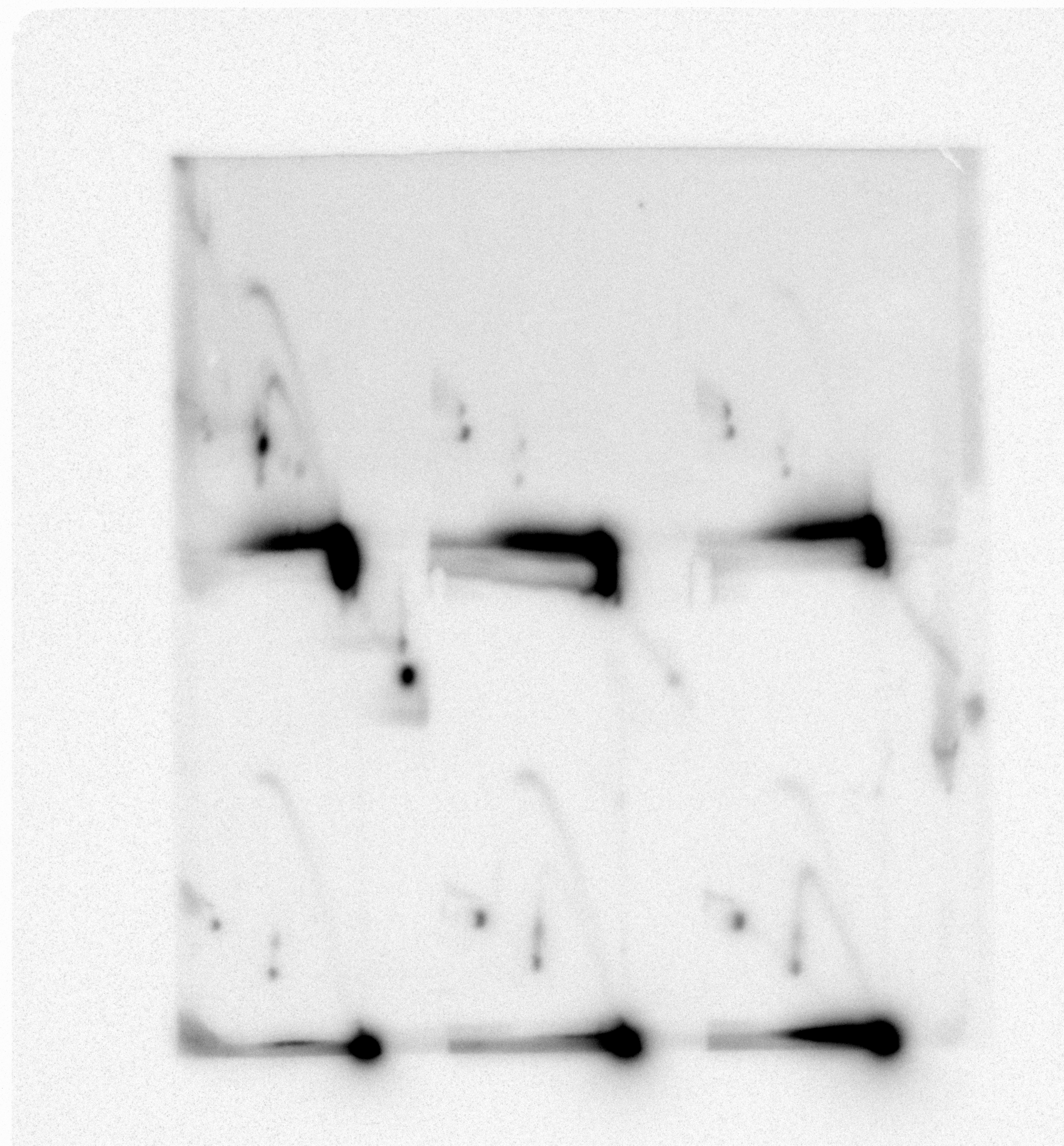

Supplement: Figure 4—source data 5. [file elife-97438-fig4-data5.zip › Figure 4 Source Data 5/rARS_WT.tif]
