# Supplementary material for: Sir2 and Fun30 regulate ribosomal DNA replication timing via MCM helicase positioning and nucleosome occupancy
Source: eLife. 2025 Jan 20;13:RP97438. doi: 10.7554/eLife.97438 (PMC11745493; doi:10.7554/eLife.97438)
Supplement: Figure 4—source data 7. [file elife-97438-fig4-data7.zip › Figure 4 Source Data 7/Figure 4F_statistical_analysis_source data.docx]

Statistical analysis of the EdU data: are the 111 early origins in Figure 4F (the EdU scatter plots) closer to WT levels in sir2 fun30 than they were in sir2. This analysis was carried out as follows:

median value in WT for 111 early origins: 6.468

median value in sir2 for 111 early origins: 6.222

median value for sir2 fun30 in 111 early origins: 6.421

p value for sir2 versus sir2 fun30: 1.298206e-40
